# Visual Results After Extended Depth-of-Focus Lens Implantation in Patients Undergoing Clear Lens Surgery

**DOI:** 10.3390/jcm14082795

**Published:** 2025-04-18

**Authors:** Emanuel Barberá-Loustaunau, Felipe Couñago, Miguel A. Sánchez-Tena, Nuria Garzón

**Affiliations:** 1 Instituto Oftalmológico Quirónsalud, P.° Marítimo 1, 15001 A Coruña, Spain; 2 Hospital Universitario Vithas La Milagrosa, Modesto Lafuente 14, 28010 Madrid, Spain; fcounago@gmail.com; 3 Hospital Universitario San Francisco de Asís, Joaquín Costa 28, 28002 Madrid, Spain; 4 Universidad Europea, Tajo, s/n, 28670 Villaviciosa de Odón, Spain; 5 Departamento de Optometría y Visión, Universidad Complutense de Madrid, Av. Arcos del Jalón 118, 28037 Madrid, Spain; masancheztena@ucm.es (M.A.S.-T.); mgarzonj@ucm.es (N.G.)

**Keywords:** extended depth-of-focus, presbyopia, refractive lens exchange

## Abstract

**Background/Objectives**: To evaluate the efficacy and visual quality provided by the extended depth-of-focus AcrySof IQ Vivity lens (Alcon Laboratories, Inc., Fort Worth, TX, USA) in patients undergoing refractive lens exchange (RLE) surgery for presbyopia correction. **Methods**: This descriptive prospective single-arm clinical study included 30 patients (60 eyes) aged 49–69 years (mean 60.2) who underwent clear lens surgery for presbyopia correction. Postoperative 3-month assessments included uncorrected distance visual acuity (UDVA), uncorrected intermediate visual acuity (UIVA), uncorrected near visual acuity (UNVA), distance-corrected intermediate visual acuity (DCIVA), and distance-corrected near visual acuity (DCNVA) measurements. Patient satisfaction and visual disturbances were evaluated using a standardized questionnaire. **Results**: Postoperative binocular visual acuity results were as follows: UDVA, 0.00 ± 0.06 logMAR; UIVA 0.08 ± 0.07 logMAR; and UNVA, 0.18 ± 0.10 logMAR. Refractive outcomes showed mean spherical equivalent values of −0.15 D ± 0.28 diopters (D) for the right eye and −0.18 D ± 0.30 D for the left eye postoperatively. Most patients (93.2%) were satisfied or very satisfied with the surgery, and 63.3% never needed glasses at any distance postoperatively. Mild and non-disabling photic phenomena were reported by 23% of patients for halos and 30% for glares. **Conclusions**: The study IOL provides excellent visual acuity for far and intermediate distances, as well as functional near vision under photopic conditions, with high levels of patient satisfaction and minimal visual disturbances. This lens is a promising option for non-cataract patients undergoing RLE for presbyopia correction.

## 1. Introduction

Intraocular lenses (IOLs) used to replace the natural lens in refractive lens exchange or cataract procedures have undergone significant advancements. Today, a wide range of IOLs is available, from advanced monofocal designs to multifocal and extended depth-of-focus lenses, which provides good vision at multiple distances. The continuous development of new IOL designs, coupled with increased life expectancy, has led to a growing number of patients seeking to reduce their dependence on glasses for vision correction.

Extended depth-of-focus (EDOF) lenses have emerged as an alternative to the traditionally used multifocal intraocular lenses (IOLs) [1]. Multifocal IOLs with two or more focal points provide good visual acuity at different distances simultaneously [2,3]. However, the transition between these focal points may cause contrast reduction and increased photic phenomena, such as halos and glare, which can ultimately affect visual quality and patient satisfaction [4,5].

The fundamental principle of EDOF IOLs is to create a single elongated focal point, which increases the depth of focus and, consequently, the range of vision. This design offers a clear vision at both far and intermediate distances, with less contrast reduction and fewer visual disturbances commonly associated with multifocal IOLs [1,6,7]. Thus, the development of EDOF lenses has accelerated owing to the increasing demand from patients to optimize postoperative visual quality at all distances and achieve spectacle independence after cataract or refractive surgery [1]. Currently, there are four main EDOF IOL technologies: small-aperture design, bioanalogic IOL, diffractive optics, and non-diffractive optical manipulation [8].

The AcrySof IQ Vivity (from now on the study IOL—Alcon, Fort Worth, TX, USA) is a recently introduced EDOF IOL that uses a non-diffractive wavefront-shaping technology [9]. To date, several studies have reported good postoperative outcomes with this lens after cataract surgery [10,11,12,13]. However, there is no evidence regarding the use of this lens in non-cataract patients undergoing clear lens surgery. Given the growing number of presbyopes who desire good visual acuity at all distances and spectacle independence, EDOF IOLs may be an effective option for these patients. The aim of this study, therefore, was to evaluate the efficacy and visual quality provided by the EDOF study IOL in patients undergoing refractive lens exchange (RLE) surgery for the correction of presbyopia.

## 2. Materials and Methods

### 2.1. Study Design

This descriptive, prospective, single-arm clinical study with a 3-month follow-up was conducted at the Instituto Oftalmológico Quirónsalud, A Coruña, Spain. Patients were required to commit to attending postoperative follow-up examinations as part of the study protocol. This study was conducted in accordance with the ethical principles of the Declaration of Helsinki and was approved by the ethics committee of the Instituto Oftalmológico Quirónsalud, A Coruña, Spain (2022/365). Patients enrolled in the study were informed of the consequences and limitations of the clinical research plan and provided written, informed consent.

### 2.2. Study Population

The participants were patients who opted for RLE surgery to correct presbyopia in the absence of cataracts. The inclusion criteria included subjects who were expected to have a postoperative refractive astigmatism of less than 0.50 D, with the possibility of implanting a toric lens. Patients with less demanding near-distance requirements and those at risk of experiencing halos, such as night-time drivers, were considered for inclusion in the study. The exclusion criteria included patients who were unable to comply with the limitations of the clinical research plan or who might not cooperate during the study period. Patients who were currently participating in other research involving drugs or health products were also excluded. Furthermore, any pathology that could significantly affect the results, such as chronic uveitis, iritis, or corneal dystrophy, resulted in exclusion from the study. Amblyopia, strabismus, diabetic retinopathy, uncontrolled glaucoma and/or intraocular pressure exceeding 24 mmHg, and age-related macular degeneration were also exclusion criteria. Patients who had undergone intraocular or corneal surgery in the past or were using systemic or ocular drug therapy that may have affected visual acuity were not eligible to participate. Finally, patients who requested independence from wearing spectacles for near vision were excluded from the study.

### 2.3. IOLs and Surgical Procedure

Patients enrolled in the study were implanted with an EDOF study IOL. The study IOL was a single-piece biconvex aspheric intraocular lens composed of a high-refractive soft acrylic material that provided blue light filtering and UV-absorbing properties [14]. The Acrysoft IQ Vivity is a C-loop single-piece IOL with a 6 mm optic and an overall diameter of 13 mm made of hydrophobic acrylic material with a refractive index of 1.55 at 35 °C and a low Abbe number of 37. The lens has an aspheric anterior surface with a central modification that includes the addition of an optical element with a toroidal profile, described by the manufacturer as a non-diffractive wavefront-shaping element (X-WAVE™ technology). The lens design incorporates two anterior surface transition elements, comprising a slightly elevated plateau (~1 µm) that stretches the wavefront, resulting in a continuously extended focal range, and a small curvature change that shifts the wavefront, ensuring that all the energy is usable. Without this shift, half of the extended focal range would be positioned in front of the retina (myopic result) and half behind it (hyperopic result), resulting in a loss of effective focal range. This small curvature change, therefore, moves the focalized range, shifting the light from the hyperopic direction to the myopic direction, ensuring that the entire light energy is effective. The two surface transition elements forming the central toroidal-shaped modification work synergistically and simultaneously to create a continuously extended focal range [15].

All surgeries were performed by the same experienced surgeon (EB) under topical anesthesia using the phacoemulsification technique, assisted by the Centurion Vision System (Alcon Laboratories, Inc., Fort Worth, TX, USA) using 2.2 mm clear corneal incisions, positioned at 45° in the left eye and 135° in the right eye for aspiration and irrigation of the cortex. The IOL was implanted into the capsular bag using a hydraulic injector via a self-sealing incision. Both eyes underwent surgery, with the second eye surgery scheduled between 1 and 2 weeks after the first eye surgery was performed. In all eyes, the IOL was implanted in the bag with the help of an injector, and all surgeries were supported by the assisted cataract surgery system (Verion Digital Marker, Alcon, Fort Worth, TX, USA) to achieve good IOL centering. All wounds were confirmed to be self-healing, and no intra-operative complications occurred. Postoperative pharmacological treatment consisted of a combination of antibiotics and steroidal anti-inflammatory drops.

### 2.4. Statistical Analysis

Statistical analysis was conducted using IBM-SPSS Statistics version 25 (IBM Corp. Released 2017. IBM-SPSS Statistics v 25.0 for Windows; Armonk, NY, USA).

A sample size of 30 patients was assumed, with a 95% confidence interval, a standard deviation of 0.1 logMAR (based on prior literature), and a precision for the UDVA estimated at 0.04 logMAR.

Quantitative variables were explored to assess their fit to a Gaussian normal distribution. The following methods were employed for these tests: (a) normal Q-Q plots, (b) skewness and kurtosis indices, (c) t-Student, and (d) the Shapiro-Wilk test for goodness-of-fit to normality, specifically designed for samples with N < 50. These variables were subsequently described using the standard measures of central tendency (mean/median) and variability (standard deviation, total range, and interquartile range).

## 3. Results

### 3.1. Patients Demographic

Sixty eyes of 30 patients underwent successful implantation of the study IOL for the correction of presbyopia. Patient ages ranged from 49 to 69 years, with a mean age of 60.2 years ± 6.0 years. Eighteen participants were women (60%). A total of 10 eyes (16.67%) required a toric IOL. Patient demographics and preoperative biometric characteristics are summarized in Table 1.

### 3.2. Visual Acuity Outcomes

The results of the postoperative examination conducted three months after RLE surgery showed a mean postoperative binocular UDVA of 0.00 ± 0.06 logMar (1.00 ± 0.14 on the decimal scale; equivalent to 20/20 Snellen). The mean binocular UIVA was 0.08 ± 0.07 logMar (0.84 ± 0.14 on the decimal scale, equivalent to 20/24 Snellen or better), and the mean binocular UNVA value was 0.18 ± 0.10 logMar (0.68 ± 0.16 on the decimal scale, equivalent to 20/30 Snellen). Table 2 presents the results of binocular and monocular visual acuities at different distances. Furthermore, only a slight improvement in intermediate and near visual acuity was observed with correction; the mean binocular DCIVA and DCNVA were 0.07 ± 0.08 logMar (equivalent to 20/23 Snellen) and 0.15 ± 0.11 logMar (equivalent to 20/28 Snellen), respectively (Table 3).

When comparing the uncorrected and corrected data at different distances, statistically significant differences were found between UIVA and DCIVA for both the right and left eyes (*p* = 0.037 and 0.0001, respectively). The same occurred for near vision in the right and left eyes (*p* = 0.010 and 0.003, respectively).

For binocular vision, no significant differences were found between the refracted and uncorrected data for intermediate or near vision (*p* = 0.280 and *p* = 0.057, respectively).

### 3.3. Refractive Outcomes

Refractive outcomes were assessed preoperatively and 3 months after RLE surgery. Preoperatively, the mean spherical equivalent value was +1.48 diopters (D) ± 2.27 D (range, −4.25 to +6.25 D) for the right eye and +1.48 D ± 2.22 D (range, −4.00 to +6.25 D) for the left eye. After 3 months of surgery, the mean spherical equivalent value was −0.15 ± 0.28 D (range, −0.50 to +0.75 D) for the right eye and −0.18 ± 0.30 D (range, −1.13 to +0.25 D) for the left eye.

### 3.4. Visual Satisfaction Outcomes

**Figure 1:**
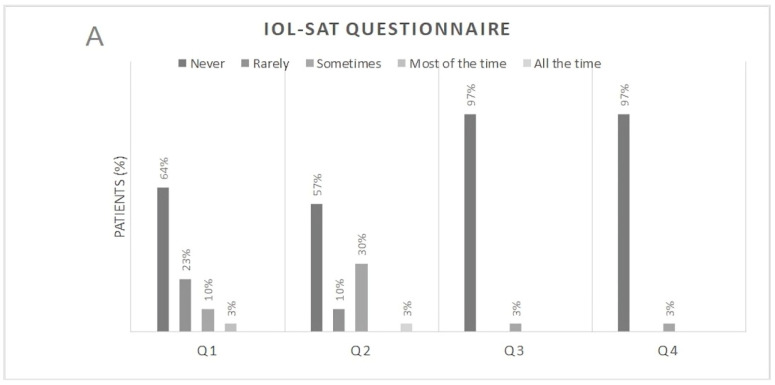

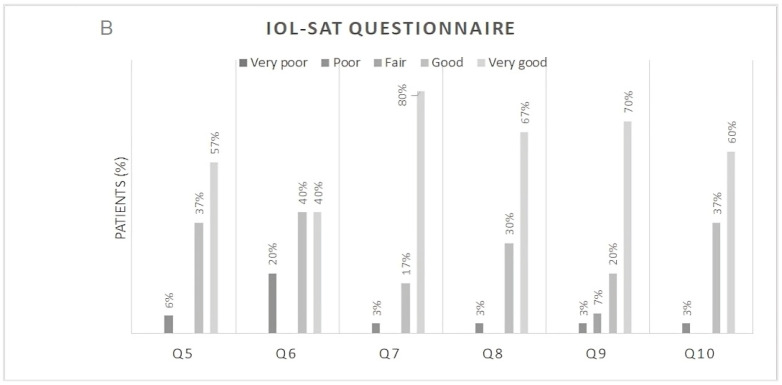
Results of the IOLSAT questionnaire at the Month-3 postoperative visit. (**A**) Q1: Overall, in the past 7 days, how often did you need to wear eyeglasses to see? Q2: In the past 7 days, how often did you need to wear eyeglasses to see “up close” (for example, reading a book)? Q3: In the past 7 days, how often did you need to wear eyeglasses to see “at arm’s-length” (for example, using an ATM or seeing the dashboard of a car)? Q4: In the past 7 days, how often did you need to wear eyeglasses to see “far away” (for example, seeing street signs)? (**B**) Q5: In the past 7 days, how often did you need to wear eyeglasses to see “up close” (for example, reading a book) in bright lighting? Q6: In the past 7 days, how often did you need to wear eyeglasses to see “up close” (for example, reading a book) in dim lighting? Q7: In the past 7 days, how often did you need to wear eyeglasses to see “at arm’s-length” (for example, using an ATM or seeing the dashboard of a car) in bright lighting? Q8: In the past 7 days, how often did you need to wear eyeglasses to see “at arm’s-length” (for example, using an ATM or seeing the dashboard of a car) in dim lighting? Q9: In the past 7 days, how often did you need to wear eyeglasses to see “far away” (for example, seeing street signs) in bright lighting? Q10: Overall, in the past 7 days, how satisfied were you with the results of the surgery?

**Figure 2:**
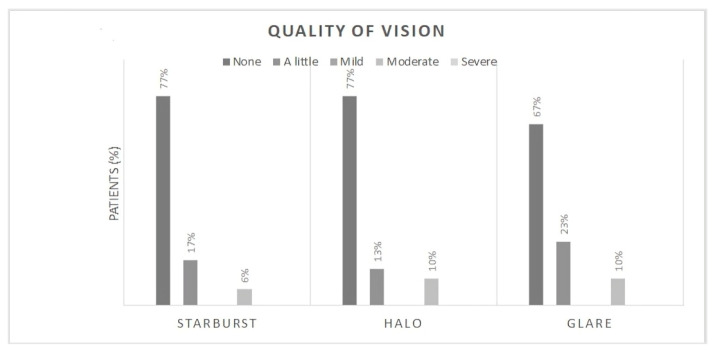
Results of the questions about quality of vision at the Month-3 postoperative visit.

Postoperative responses of patients to the IOLSAT (Figure 1) and QUVID (Figure 2) questionnaires at the 3-month follow-up. Specifically, 87% of the patients never or rarely needed glasses at any distance, while 13% needed them sometimes or most of the time. Twenty-nine patients reported never needing glasses for either far or intermediate distances. Moreover, 94% of the participants rated their near vision as either good or very good under bright lighting conditions and 97% under the same conditions at intermediate distance. Under dim lighting conditions, the values were 80 and 97%, respectively. The 97% of the patients considered the results obtained after surgery to be very good or good. In relation to photic phenomena, 83% of patients reported experiencing no or minimal halos, 94% reported no or minimal starbursts, and 90% reported no or minimal glare.

No adverse events were reported in this study.

## 4. Discussion

Independence from glasses after cataract or refractive surgery is becoming increasingly important to patients; therefore, the need to rely on glasses for near vision after surgery can lead to patient dissatisfaction [2,4]. These requirements suggest that EDOF lenses may not be an optimal solution for the correction of presbyopia. With this in mind, we aimed to evaluate the visual outcomes of patients with presbyopia who opted for clear lens surgery with the AcrySof IQ Vivity IOL.

Postoperative examination conducted 3 months after RLE surgery showed satisfactory visual acuity results at both far and intermediate distances, as well as good functional near visual acuity. The refractive results also showed a significant improvement after surgery, demonstrating the effectiveness of the procedure in achieving refractive correction and improving the overall visual performance. Several studies have reported that the near vision provided by EDOF lenses, including the study IOL, is not as good as that provided by multifocal lenses; therefore, spectacles are required to optimize near vision acuity [10,11,16,17]. However, other studies have demonstrated that this lens provides functional near vision acuity, enabling patients to live their daily lives without glasses [9,12,18]. Our results confirm that the study IOL not only provides good visual acuity at both far and intermediate distances but also allows good functional near visual acuity under photopic conditions. Furthermore, our results complement those of previous studies conducted in patients undergoing cataract surgery [9,12,18], since our findings indicate that patients opting for RLE may also benefit from EDOF lenses.

The majority of our patients expressed highly favorable responses to the quality of vision questionnaires administered three months after surgery. 97% of patients reported that they never required spectacles for distance or intermediate vision, and the majority rated their near vision as good or very good. Indeed, more than half of the patients stated that they never required spectacles for near vision. When comparing the results with and without correction for each eye, statistically significant differences were observed for both intermediate and near vision. However, these values were not clinically relevant, as they corresponded to a difference of only 2 or 3 letters in all cases.

Previous studies have already used questionnaires to assess the quality of vision and spectacle independence, reporting good postoperative outcomes with the study IOL [9,10,13]. However, to our knowledge, this is the first study to evaluate the visual outcomes of this EDOF lens in non-cataract patients undergoing RLE for the correction of presbyopia. The results reported by Bansal et al. [19], who studied the same lens in patients with cataracts, showed that spectacle independence was achieved in 100%, 94.7%, and 94.7% of patients for distance, intermediate, and near vision, respectively, at three months. These findings are very similar to those of our study for distance and intermediate vision, with slightly better outcomes for near vision.

The experience of photic phenomena is quite common in patients receiving multifocal IOLs. These phenomena, including halos, glare, and starbursts, can be considered a potential limitation for patients, particularly in low-light conditions, such as night driving [4,10]. EDOF lenses have been shown to induce fewer visual disturbances than multifocal lenses [6,7]. Indeed, non-diffractive EDOF IOLs, such as the study IOL, have demonstrated a significantly reduced incidence of optical phenomena compared to multifocal lenses or other EDOF technologies and are comparable to monofocal IOLs [9,13,20,21]. Furthermore, Guarro et al. concluded that non-diffractive EDOF lenses in particular should be considered for individuals in whom the presence of these visual disturbances could considerably impact their quality of life, such as night-time drivers or individuals with large pupils [20].

Results from the questionnaires completed by patients following RLE indicated that the percentage of patients reporting visual disturbances was relatively low when the EDOF lens was implanted for the correction of presbyopia. In fact, 23% of patients reported a little glare, and 10% reported moderate glare, which is in line with Arrigo et al., who reported on real-life experience with the lens three months after cataract surgery [10]. The percentage of patients reporting halos in our study was lower than that reported by Arrigo et al., but similar to the rates reported by Guarro et al. [20]. In addition, patients in our study who reported visual disturbances described them as mild and not particularly bothersome.

Choi et al. [22], using a diffractive EDOF lens, reported that in a non-directed survey, 23.0% of patients experienced some degree of dysphotopsia. Overall, 92.0%, 89.4%, and 86.7% of patients reported no or minimal halos, glare, and starbursts, respectively. Tañá-Sanz et al. [23], using the Asqelio™ EDOF IOL—a lens with a Phase-Ring™-structured design on its posterior surface—reported no significant visual symptoms in terms of frequency, intensity, or bothersomeness following implantation. In that study, halos were the only relevant visual symptom, with 18.19% of patients reporting their presence quite often or very often, but none experienced severe bother. In both cases, the patients had cataracts prior to surgery.

In our study, the questionnaire results indicated that the vast majority of participants were very satisfied or satisfied with the surgical intervention. It is important to note that the outcomes of the questionnaires could be highly influenced by patient expectations of a clear crystalline lens, which tend to be higher in younger patients. Accordingly, the lower incidence of photic phenomena observed with the AcrySof IQ Vivity lens, coupled with its ability to provide excellent vision at both far and intermediate distances, as well as functional near vision, suggests that this EDOF lens is potentially a favorable choice for patients undergoing clear lens surgery.

One of the limitations of this study is that we decided not to perform a dedicated analysis of eyes that required toric lenses, due to the relatively low number of participants in this subgroup. However, previous studies have demonstrated that Vivity toric IOL implanted in eyes with low astigmatism provide accurate refractive outcomes, good visual acuity at different distances, and excellent rotational stability in patients undergoing cataract surgery [12,24].

The robustness of our findings could also be enhanced by conducting larger multicenter studies involving a greater number of patients undergoing clear lens surgery for presbyopia correction and other refractive errors.

## 5. Conclusions

In conclusion, the study AcrySof IQ Vivity IOL provides good visual acuity for both far and intermediate distances while allowing good functional near vision under photopic conditions in patients undergoing RLE for the correction of presbyopia. High levels of satisfaction and independence from glasses were achieved, although glasses may be needed for near use under certain low-light conditions. The photic phenomena were well tolerated. These factors suggest that the AcrySof IQ Vivity lens may be a promising option for patients undergoing clear lens surgery.

## Figures and Tables

**Table 1:** Demographic data of the patients and biometric data before clear lens surgery.

Patient Demographics and Biometric Characteristics (*n* = 30)	
Median age, years (range)	60.2 (49, 69)
Sex, *n* (%)	
Women	18 (60)
Men	12 (40)
Toric IOL, *n* eyes (%)	10 (16.67)
IOL power (D), mean ± SD (range)	
Right eye	23.1 ± 3.3 (16, 29.5)
Left eye	23.4 ± 3.3 (17.5, 30)
Axial length (mm), mean ± SD (range)	
Right eye	23.3 ± 1.2 (21.4, 26.1)
Left eye	23.3 ± 1.3 (21.3, 26.2)
Keratometry (D), mean ± SD (range)	
Right eye	42.9 ± 1.5 (39.1, 45.8)
Left eye	43.0 ± 1.5 (39.8, 46.0)

D = diopters; SD = standard deviation.

**Table 2:** Distance (UDVA), intermediate (UIVA), and near (UNVA) visual acuity 3 months postoperatively (*n* = 60 eyes).

	Mean ± SD	Median (IQR)
UDVA—Binocular	0.00 ± 0.06	0.00 (0.07)
UDVA—Right eye	0.06 ± 0.08	0.05 (0.10)
UDVA—Left eye	0.10 ± 0.11	0.05 (0.15)
UIVA—Binocular	0.08 ± 0.07	0.10 (0.10)
UIVA—Right eye	0.17 ± 0.13	0.20 (0.12)
UIVA—Left eye	0.18 ± 0.10	0.20 (0.13)
UNVA—Binocular	0.18 ± 0.10	0.20 (0.12)
UNVA—Right eye	0.34 ± 0.13	0.30 (0.20)
UNVA—Left eye	0.32 ± 0.15	0.30 (0.20)

IQR = interquartile range; SD= standard deviation; UDVA = uncorrected distance visual acuity; UIVA = uncorrected intermediate visual acuity; UNVA = uncorrected near visual acuity. The mean/median values are represented on the logMAR scale.

**Table 3:** Visual acuity values three months postoperatively with distance correction (*n* = 60 eyes).

	**Mean ± SD**	**Median (** **IQR)**
DCIVA—Binocular	0.07 ± 0.08	0.10 (0.10)
DCIVA—Right eye	0.14 ± 0.09	0.10 (0.10)
DCIVA—Left eye	0.14 ± 0.11	0.10 (0.12)
DCNVA—Binocular	0.15 ± 0.11	0.10 (0.12)
DCNVA—Right eye	0.27 ± 0.16	0.30 (0.30)
DCNVA—Left eye	0.23 ± 0.14	0.20 (0.20)

DCIVA = distance-corrected intermediate visual acuity; DCNVA = distance-corrected near visual acuity; IQR = interquartile range; SD = standard deviation. The mean/median values were represented on the logMAR scale.

## Data Availability

The data obtained in this study can be requested from the research team by contacting the corresponding author.

## Abbreviations

The following abbreviations are used in this manuscript:

CDVA	Corrected distance visual acuity
D	Diopter
DCIVA	Distance-corrected intermediate visual acuity
DCNVA	Distance-corrected near visual acuity
EDOF	Extended depth-of-focus
IOL	Intraocular lens
IOLSAT	Intraocular Lens Satisfaction
QVID	Questionnaire for Visual Disturbances
RLE	Refractive lens exchange
UDVA	Uncorrected distance visual acuity
UIVA	Uncorrected intermediate visual acuity
UNVA	Uncorrected near visual acuity
VA	Visual acuity
